# Cluster analysis of patients with granulomatosis with polyangiitis (GPA) based on clinical presentation symptoms: a UK population-based cohort study

**DOI:** 10.1186/s13075-022-02885-9

**Published:** 2022-08-19

**Authors:** Rasiah Thayakaran, Ruchika Goel, Nicola J. Adderley, Joht Singh Chandan, Dawit Zemedikun, Krishnarajah Nirantharakumar, Lorraine Harper

**Affiliations:** 1 University of Birmingham, Institute of Applied Health Research, Birmingham, B15 2TT UK; 2 Christian Medical College, Vellore, India; 3 Queen Elizabeth Hospital Birmingham, Institute of Clinical Sciences, Centre for Translational Inflammation Research, Mindelsohn Way, Edgbaston, Birmingham, B15 2WB UK

**Keywords:** Granulomatosis with polyangiitis, Wegener’s granulomatosis, Primary care, Symptoms, Cluster analysis

## Abstract

**Background:**

Granulomatosis with polyangiitis (GPA) is small vessel vasculitis with heterogeneous clinical presentation. In the present population-based cohort study, we classified patients with GPA based on clinical features at presentation using an unsupervised clustering approach and compared their mortality, infections and frequency of comorbidities.

**Methods:**

In this open cohort study, de-identified primary care data of patients with GPA included in the IQVIA Medical Research Data database between 1 January 1995 and 25 September 2019 was analysed retrospectively. Latent class analysis was performed to create symptom clusters of patients based on 16 categories of symptoms representing various organ involvement. All-cause mortality of resultant clusters was compared after adjusting for age, sex, Townsend deprivation quintile and smoking status at index date using extended Cox proportional hazards models. Prescription of antibiotics, considered as an indirect indicator of recurrent bacterial infection, was compared using a recurrent event model, after adjusting for quarterly use of steroid as a time-dependent covariate. Cumulative frequencies of common comorbidities were compared among the clusters at index visit, 1-year and 3-year follow-up.

**Results:**

Altogether, 649 patients with GPA [median age 60.0 (IQR: 49.6–70.1)] were included. Three clusters were identified: patients with limited disease mainly with involvement of ENT and cough were classified into cluster 1 (*n* = 426); cluster 2 had generalised non-renal disease (*n* = 176); while patients in cluster 3 had renal-predominant disease (*n* = 47). Many patients in cluster 1 developed generalised disease at the end of 1 year. Mortality in clusters 2 and 3 was higher compared with cluster 1. Mortality in cluster 1 itself was 68% higher than the general population without GPA. The duration of antibiotics prescription and frequency of coexisting medical illnesses was also higher in clusters 2 and 3.

**Conclusions:**

In a primary care setting, patients with GPA can be classified into three distinct clusters with different prognosis, susceptibility to recurrent infections and presence of comorbidities. The tendency of cluster 1 to evolve into a more generalised disease raises questions about current immunosuppressive treatment approaches in these patients.

**Supplementary Information:**

The online version contains supplementary material available at 10.1186/s13075-022-02885-9.

## Background

Anti-neutrophil cytoplasmic antibodies (AAV)-associated vasculitis is a heterogeneous group of diseases characterised by inflammation of small blood vessels. Based on clinical phenotype, AAV is classified into 3 broad overlapping diseases: granulomatosis with polyangiitis (GPA), microscopic polyangiitis (MPA) and eosinophilic granulomatosis with polyangiitis (EGPA) [1]. The treatment strategy and prognosis vary according to organ involvement. Attempts have been made to classify patients with AAV using clustering data-driven approaches [2–4]. GPA by itself is a heterogeneous entity with a myriad of clinical presentations ranging from limited otolaryngeal (ENT) disease to renal limited disease and a generalised phenotype that warrants a formal data-driven classification [5, 6]. Although patients with GPA have been empirically sub-divided into subtypes for the purpose of inclusion in clinical trials, a formal clinically and prognostically meaningful classification based on clinical features indicating all the organs involved would provide uniformity [3].

Clustering is a statistical method that classifies disease into different homogenous subtypes based on clinical similarities. The resulting clusters not only identify phenotypic groups but more importantly may guide prognostication of disease and tailoring of immunosuppression in patients with GPA.

In the present population-based cohort study, we aimed to classify patients with GPA based on clinical features at presentation using an unsupervised clustering approach. We also determined the impact of these clusters on mortality. As infections and coexisting illnesses are among the key determinants of mortality in GPA, we also analysed antibiotics usage in resultant clusters as a surrogate marker of bacterial infection and explored frequency of comorbidities [7, 8].

## Methods

### Study design and data source

This study is a population based, retrospective open cohort study using the IQVIA Medical Research Data (IMRD) database. The study period was between 1 January 1995 and 25 September 2019. The primary study aim was to create symptom clusters of patients diagnosed with GPA using latent class analysis (LCA).

IMRD, comprising data from The Health Improvement Network (THIN), is a UK electronic primary care records database consisting of anonymised medical records for 15.8 million patients from 808 general practices using Vision electronic medical record software. Information on patient demographics, symptoms, diagnoses and prescriptions is included. Symptoms and diagnoses are stored as Read codes (seven character string variables which are hierarchical in nature) [9]. All prescriptions issued in primary care are recorded in IMRD. It is broadly representative of the UK population in terms of demographics, disease prevalence and mortality rates [10].

Data quality was maximised by including general practices from the latest of 1 year after they began using Vision software and 1 year after their acceptable mortality reporting (AMR) date [11, 12]. The acceptable mortality reporting date for each practice is when the practice publishes mortality rates similar to the expected rate for their population outlined by the Office for National Statistics (ONS) [13]. Patients were included 1 year following their registration, to allow time for general practices to upload historic records prior to their transfer.

### Study population and clinical variables

Patients aged 12 years and above with an incident clinically (Read) coded diagnosis (diagnosis was recorded during the eligible study period) of GPA/Wegener’s Granulomatosis were included. The clinical features pertaining to GPA were identified at the index date (GPA diagnosis date) using Read codes; these aligned with those suggested in a previous population study on GPA (Read code lists available from corresponding author upon request) [14, 15]. The clinical features used for clustering algorithms were grouped into 16 categories based on organ involvement (Table 1). Symptom categories were eye; ear, nose and throat (ENT); cough; breathlessness; acute renal failure (ARF); chronic kidney disease (CKD); presence of proteinuria or haematuria; musculoskeletal; constitutional; fatigue; dermatological; angina pain reflecting cardiac involvement; non-specific chest pain of other aetiology; specific gastrointestinal (GI) symptoms including bloody diarrhoea, abdominal pain and distension; non-specific abdominal symptoms; and neuropathy.

**Table 1:** Description of presenting clinical features used in cluster analysis

	Description
Eye	Keratoconjunctivitis, episcleritis, scleritis, uveitis, non-infective conjunctivitis, significant proptosis without underlying infection or malignancy
ENT	Deafness, blocked ear, otitis media, ear discharge, mastoiditis, sinusitis with symptoms of blocked sinus and sinus pain or deformity, purulent or bloody nasal discharge, nasal ulceration as evidenced by epistaxis, congestion, discharge or obstruction, deafness (hearing difficulty, deafness, blocked ear, deteriorating hearing without specified cause), hoarseness of voice and subglottic stenosis
Cough	Any cough
Breathlessness	Breathlessness, shortness of breath, dyspnoea of any severity and aetiology
Cardiac chest pain	Cardiac chest pain, angina
Chest pain (not specified)	Chest pain other than cardiac chest pain and angina
Chronic renal disease (CRF)	Chronic renal failure, chronic renal disease (underlying cause hypertension)
Acute renal failure (ARF)	Acute renal failure, raised creatinine, acute or chronic renal failure
Proteinuria/haematuria	Proteinuria, haematuria (any aetiology)
Fatigue	Any fatigue, lethargy, malaise, asthenia, tiredness, lassitude, general weakness
Constitutional	Fever/sweats (fevers, pyrexia, rigours or night sweats without specified cause), lethargy and documented weight loss due to any cause
Musculoskeletal	Myalgia (aching muscles, muscle pain, polymyalgia), arthritis (arthralgia or arthritis without specified cause)
Neuropathy	Peripheral neuropathy, tingling or burning sensation of extremity
Rash	Any rash or purpura
Gastrointestinal	Abdominal symptoms of intestinal aetiology including abdominal pain, bleeding in stools and abdominal distension
Non-specific abdominal symptoms	Diarrhoea of any aetiology, abdominal pain due to other causes and bloating

Patients were followed up from the date of GPA diagnosis (index date) until the exit date, defined as the first of the following events: diagnosis of outcome of interest, death, the date the patient left the practice, the last data collection date from the GP practice and the study end date. All antibiotic prescriptions were noted between index and study exit date. In instances where a patient was prescribed antibiotics more than once within a 3-month period, all the entries during that period were considered as one single prescription for analysis.

### Outcomes

All-cause mortality of resultant clusters was compared after adjusting for age (continuous), sex, Townsend deprivation score quintile [16] and smoking status categorised as smoker or non-smoker at index date. Post hoc, mortality in the low disease burden cluster was compared with matched controls without GPA to determine whether mortality was higher in this cluster than in the general population. The prescription of antibiotics which was considered as an indirect indicator of recurrent bacterial infection was also compared among GPA clusters. The date of the last recorded antibiotic prescription was considered as exit date for antibiotic prescription analysis. Data related to prescription of any antibiotic except oral co-trimoxazole, which is primarily given as prophylaxis to prevent nasal relapses in AAV, was extracted using drug codes. All antibiotics except oral co-trimoxazole were included. In addition to the standard covariates (age, sex, smoking and Townsend quintile), the results were also adjusted for quarterly use of steroids as a time dependent covariate. Additionally, cumulative frequencies of comorbidities which are commonly described in patients with AAV were compared among the three resultant clusters at index visit and 1-year and 3-year follow-up periods.

### Statistical analysis

Categorical data at index visit were described using number and proportion, while means with standard deviations (SD) or median with interquartile range (IQR) were used to describe continuous data. Missing data for covariates was included in the final analysis as a separate category.

#### Cluster analysis methodology

Cluster analysis was performed using latent class analysis (LCA) which is an unsupervised learning technique for dividing a set of members/patients into hidden classes or clusters of similar characteristics, based on observed continuous and categorical variables. Mathematically, it takes the form of a finite mixture model and is estimated by expectation-maximization (EM) procedure, similar to the K-means algorithm. The method assumes conditional independence of indicators given latent class membership, while no assumptions related to parametric distributions, linearity, normal distribution or homogeneity are made. In this study, we used the R package poLCA to perform estimation of latent class membership based on clinical features [17]. The estimation of latent classes can be sensitive to particular choices of randomised starting points if a local rather than a global maximum likelihood is reached; hence the algorithm was run 30 times to ensure stability. The optimal number of clusters was chosen using Akaike’s Bayesian information criterion (ABIC). The clinical meaningfulness and predictive value of clusters was checked before deciding the final number of clusters. The results were validated by statistical bootstrapping criteria for cluster stability (Jaccard index) against more traditional cluster analysis methods and criteria for causally reasonable medical co-associations.

#### Cox regression

An extended Cox proportional hazards model was used to estimate hazard ratios and their corresponding 95% confidence intervals for both outcomes (mortality and antibiotic use) [18] in the resultant clusters. Steroid use during every 3-month period was incorporated as a time-varying categorical covariate, categorised as follows: cumulative steroid use for ≤ 4 quarters, between 5 and 12 quarters and >12 quarters. Antibiotic use during each 3-month interval (quarters) was considered as a recurrent event. Crude/unadjusted hazard ratios were calculated with clusters of symptoms considered as a covariate. Fully adjusted hazard ratios were calculated, adjusting for clusters of symptoms, age, sex, smoking status and Townsend deprivation quintile, with cumulative steroid use as a time-varying covariate.

## Results

### Baseline demographic and clinical characteristics of patient clusters with GPA

Altogether, 649 patients with GPA, with median age 60.0 (IQR: 49.6–70.1) years, and 350 (50.9%) males were identified. Cluster analysis of patients using the 16 clinical features as described in the above methods revealed a three-cluster model as the best model with minimum aBIC. The presenting features of the entire cohort and the three clusters at index visit are compared in Table 2.

**Table 2:** Baseline demographic and clinical characteristics of patient clusters with GPA

	All patients	Cluster 1	Cluster 2	Cluster 3	*p* value^a^
**Number of patients**	649	426	176	47	-
**Median (IQR) age, years**	60.0 (49.6- 70.1)	58.5 (47.8- 67.3)	63.5 (53.7-74.2)	68.9 (57.4-79.5)	<0.001
**Gender Females,*n*(%)**	350 (53.9)	245 (57.5)	82 (46.6)	23 (48.9)	0.039
**Smoking history,*n*(%)**	293 (48.0)	186 (47.3)	86 (50.0)	21 (46.7)	0.828
** Missing**	39 (6.0)	33 (7.7)	4 (2.3)	2 (4.3)	
**Townsend quintile,*n*(%)**					
**1 (least deprived)**	151 (23.3)	107 (25.1)	33 (18.8)	11 (23.4)	0.155
**2**	130 (20.0)	83 (19.5)	37 (21.0)	10 (21.3)	
**3**	100 (15.4)	83 (19.5)	40 (22.7)	7 (14.9)	
**4**	100 (15.4)	63 (14.8)	28 (15.9)	9 (19.1)	
**5**	61 (9.4)	33 (7.7)	25 (14.2)	3 (6.4)	
**Missing**	107 (16.5)	57 (13.4)	13 (7.4)	7 (14.9)	
**Eye**	120 (18.5)	58 (13.6)	46 (26.0)	16 (34.0)	< 0.001
**ENT**	385 (59.3)	211 (49.5)	157 (89.2)	17 (36.2)	< 0.001
**Cough**	317 (48.8)	150 (35.2)	135 (76.7)	32 (68.1)	< 0.001
**Breathlessness**	134 (20.6)	35 (8.2)	82 (46.6)	17 (36.2)	< 0.001
**Cardiovascular (Chest pain/angina)**	32 (4.9)	7 (1.6)	18 (10.2)	7 (14.9)	< 0.001
**Chest pain (not specified)**	189 (29.1)	76 (17.8)	102 (58.0)	11 (23.4)	< 0.001
**CKD**	95 (14.6)	22 (5.2)	35 (19.9)	38 (80.9)	< 0.001
**ARF**	50 (7.7)	11 (2.6)	11 (6.2)	28 (59.6)	< 0.001
**Proteinuria/haematuria**	49 (7.6)	25 (5.9)	8 (4.5)	16 (34.0)	< 0.001
**Fatigue**	137 (21.1)	53 (12.4)	68 (38.6)	16 (34.0)	< 0.001
**Constitutional**	36 (5.5)	11 (2.6)	21 (11.9)	4 (11.1)	< 0.001
**Musculoskeletal**	182 (28.0)	77 (18.1)	83 (47.2)	22 (46.8)	< 0.001
**Neuropathy**	42 (6.5)	20 (4.7)	20 (11.4)	2 (4.3)	0.008
**Dermatological (Rash)**	188 (29.0)	61 (14.3)	117 (66.5)	10 (21.3)	< 0.001
**Gastrointestinal**	92 (14.2)	27 (6.3)	65 (36.9)	0 (0.00)	< 0.001
**Non-specific abdominal symptoms**	130 (20.0)	36 (8.5)	93 (52.8)	1 (2.1)	< 0.001

^a^For comparison of the three clusters. *ARF* acute renal failure, *CKD* chronic kidney disease, *ENT* ear, nose, throat, *IQR* interquartile range

ENT symptoms and cough were commonly present in all three clusters, with the highest frequency in cluster 2 (89.2% and 76.7%, respectively). Patients classified into cluster 1 presented with symptoms relatively limited to ENT involvement (59.3%) and cough (48.8%) at the index visit. Symptoms suggestive of generalised disease including angina (1.6%), ARF (2.6%), CKD (5.2%), fatigue (12.4%), non-specific chest pain (17.8%) and musculoskeletal symptoms (18.1%) were less frequent in this cluster at the index visit. Cluster 2 comprised patients presenting with widespread extra-renal disease including symptoms suggestive of gastrointestinal (36.9%), musculoskeletal (47.2%), dermatological (skin rash, 66.5%), ENT (89.2%) and respiratory involvement (cough in 76.7% and breathlessness in 46.6%). However, cluster 2 had a relatively low frequency of CKD, ARF and urinary abnormalities (19.9%, 6.2% and 4.5%, respectively) that differentiated it from patients categorised into cluster 3, who presented with renal predominant disease with high frequency of ARF, CKD and urinary abnormalities (59.6%, 80.9% and 34.0%, respectively). Extra-renal involvement including symptoms suggestive of gastrointestinal, cardio-vascular and dermatological disease were less common in cluster 3 (0%, 14.9% and 21.3%, respectively) compared with cluster 2.

### Evolution of clusters over 3 years

New symptoms accrued by patients in the three clusters were analysed at 1-year and 3-year follow-up. Many of the patients in cluster 1 evolved symptoms suggestive of systemic disease including, dermatological (*n* = 105, 24.6%), musculoskeletal (*n* = 85, 19.9%), eye (*n* = 81, 19.0%) involvement, non-specific abdominal complaints (*n* = 76, 17.8%) and gastrointestinal involvement (*n* = 69, 16.2%) at the end of 1 year of follow-up. A smaller number developed renal involvement in the form of ARF (*n* = 37, 8.7%), CKD (*n* = 25, 5.9%) and proteinuria (*n* = 32, 7.5%) and symptoms suggestive of cardiovascular involvement (*n* = 23, 5.4%). New onset cough, dyspnoea and ENT involvement was recorded in 124 (29.1%), 79 (18.5%) and 110 (25.8%) patients, respectively, while 22 (5.2%) developed features of neuropathy. Another 28 and 8 patients in cluster 1 developed CKD and proteinuria at the end of 3 years of follow-up. Less than 25% of patients in cluster 3 developed symptoms suggestive of extra-renal involvement (Supplementary table 1). During the entire follow-up, patients in cluster 1 received a median of 9 (IQR 3-21) quarters of steroids which was higher than steroid use in cluster 2 [6 (2–15) quarters] and cluster 3 [7 (2–16) quarters], (*p* = 0.031). During the first 3 years after index visit, patients in clusters 1, 2 and 3 received steroids for 9 (3–12), 6 (2–12) and 7 (2–12) quarters respectively, *p* = 0.048.

### Outcome analysis of clusters

Distinct differences in mortality and antibiotic prescriptions, which was indirect evidence of bacterial infections, were observed among the three clusters. As the frequency of these outcomes was the least in cluster 1, it was chosen as the reference cluster for analysis.

#### Mortality

**Fig. 1:**
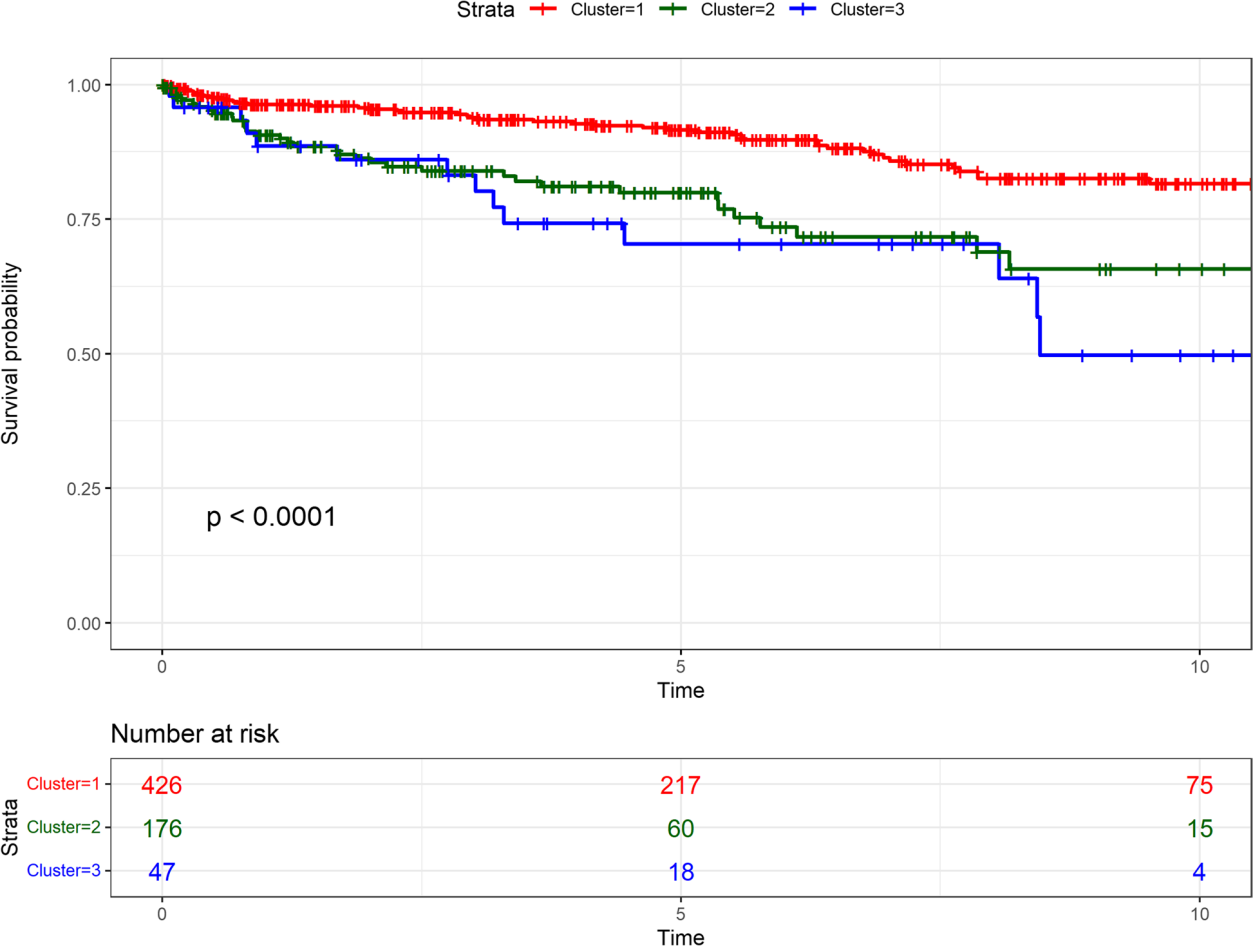
Mortality survival plot for the three clusters of patients with GPA

Overall, there were 103 fatalities during the median follow-up period of 4.45 (IQR 1.59–7.78) years. Compared to cluster 1, mortality was significantly higher in both cluster 2 with widespread extra-renal disease (aHR 1.61 (95% CI 1.03–2.53, *p* = 0.037)) and cluster 3 (1.95 (1.05–3.62, *p* = 0.035)), after adjusting for age, gender, smoking and Townsend index and steroid use (Table 3, Fig. 1). Increasing age, male gender and Townsend quintile 5 (most deprived) also predicted increased overall mortality over time, with an aHR of 1.08 (95% CI 1.06–1.11, *p* < 0.001), 2.33 (1.50–3.60, *p* < 0.001) and 3.44 (1.78–6.62, *p* < 0.001), respectively. Steroid use analysed as a time dependent covariate was not associated with mortality. Although mortality risk was the lowest in cluster 1, it was still higher than an age-matched control population, with an aHR of 1.68 (1.16–2.42, *p* < 0.01) (Supplementary table 2). Among presenting clinical features at index visit, breathlessness and cardiac chest pain predicted increased mortality with an aHR of 3.04 (1.86–4.96, *p* < 0.001) and 1.96 (1.05–3.67, *p* = 0.04), respectively.

**Table 3:** Mortality and antibiotic usage in clusters of patients with GPA

	Cluster 1 (***n*** = 426) (limited disease)	Cluster 2 (***n*** = 176) (extra-renal generalised disease)	Cluster 3 (***n*** = 47) (renal predominant disease)
**Mortality**			
No. of deaths (%)	52 (12.2%)	37 (21%)	14 (29.8%)
PY, median (IQR)	5.14 (2.00–8.69)	3.35 (1.05–5.75)	3.68 (1.34–7.72)
Unadjusted HR (95% CI), *p* value	1 (reference)	2.33 (1.53–3.57), *p* < 0.001	2.95 (1.63–5.33), *p* < 0.001
aHR (95% CI), *p* value^a^	1 (reference)	1.61 (1.03–2.53), *p* = 0.037	1.95 (1.05–3.62), *p* = 0.035
**Antibiotics use**			
Cumulative period of use of antibiotics, in quarters of year (total number of antibiotic prescriptions)	3844 (9606)	596 (1636)	1339 (2874)
Unadjusted HR (95% CI), *p* value	1 (reference)	1.64 (1.52–1.77), *p* < 0.001	1.15 (1.00–1.33), *p* = 0.04
aHR (95% CI), *p* value^a^	1 (reference)	1.46 (1.35–1.58), *p* < 0.001	1.21 (1.05–1.39), *p* = 0.009

^a^Adjusted hazard ratio (aHR) was calculated after adjusting for age, sex, Townsend deprivation quintile, smoking and duration of steroid use. *PY* person years

**Fig. 2:**
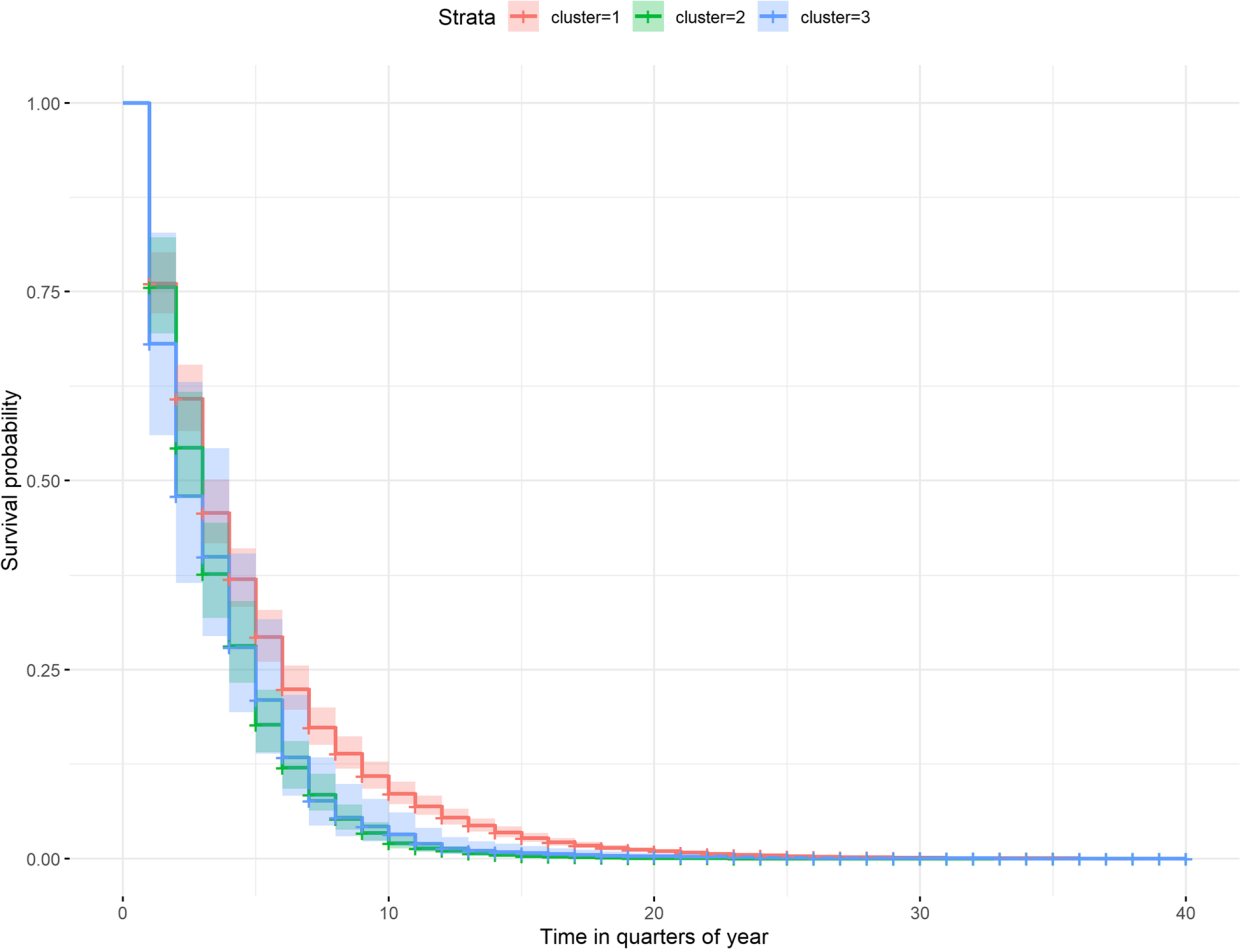
Cumulative risk of antibiotic use in clusters of patients with granulomatosis with polyangiitis

Cause of death was unavailable in the routinely collected dataset used. Other studies have shown that infection is a risk factor for death. We therefore used antibiotic prescriptions as a surrogate for infection. Overall, 5779 three-monthly prescriptions of antibiotics were observed in the entire cohort. The risk of recurrent use of antibiotics measured as a single event every quarter was significantly increased by 1.46 and 1.21 fold in cluster 2 and cluster 3, respectively, when compared with cluster 1, *p* < 0.001 and *p* = 0.009, respectively (Fig. 2, Table 3). Furthermore, the hazard of antibiotic use was higher in patients classified in Townsend class 2, 3, 4 or 5 as compared with Townsend class 1 (least deprived), with an aHR of 1.21 (95% CI 1.09–1.34, *p* < 0.001), 1.12 (1.00–1.24, *p* = 0.04), 1.41 (1.27–1.57, *p* < 0.001) and 1.65 (1.45–1.87, *p* < 0.001), respectively. Male sex and increasing age were protective against antibiotic use with an aHR of 0.62 (0.58–0.66, *p* < 0.001) and 0.99 (0.99–0.99, *p* < 0.001) per year, while smoking was associated with increased risk of antibiotic use (1.13 (1.05–1.22, *p* < 0.001)). The risk was also higher for patients who received a prescription of steroids for 5 to 12 quarters (1.18 (1.06–1.32, *p* = 0.003)) and >12 quarters (1.52 (1.35–1.71, *p* < 0.001)) compared with patients who were prescribed steroids for < 5 quarters.

#### Comorbidities

**Fig. 3:**
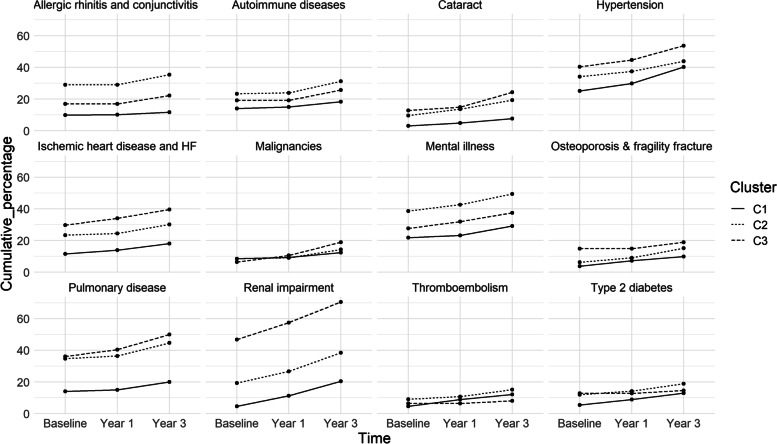
Comorbidities at baseline and at 1- and 3-year follow-up for the three clusters of patients with GPA based on symptom clusters

Overall, cluster 1 had the smallest number of comorbidities at presentation and at 3 years’ follow-up. Hypertension, mental illness and ischaemic heart disease were the most common comorbidities in all three clusters at index visit as well as at the end of the 3-year follow-up (Fig. 3). In addition, pulmonary disorders were present in >30% of patients in clusters 2 and 3 at index visit, which increased to > 40% at 3 years (Fig. 3).

## Discussion

In this first study to sub-categorise GPA using a data-driven approach, our primary aim was to discover meaningful, causally associated and medically predictive clusters of patients in a high-quality database. Three clinically meaningful distinct clusters of patients were identified. Patients in cluster 1 with limited disease phenotype, presenting mainly with involvement of ENT and cough, had remarkably better prognosis in terms of mortality and use of recurrent antibiotics compared with patients presenting with generalised non-renal disease (cluster 2) and renal predominant disease (cluster 3). Interestingly, patients with the limited disease phenotype received longer duration of steroids during follow-up and a sizeable proportion of them accrued new symptoms suggestive of generalised disease as early as after 1 year of follow-up. A plausible explanation for longer use of steroids in cluster 1 could be more relapses due to inadequate treatment which was not captured due to limited information in the database. This suggests a need for closer monitoring of this subgroup of GPA so that immunosuppression can be modified at early signs of relapse or disease evolution before organ damage ensues. This also implies a potential need to consider a treatment strategy of more aggressive initial treatment for this subgroup of GPA.

Despite different population settings, the results of this study are similar to clustering observed in patients with ANCA vasculitis recruited to EUVAS clinical trials [2]. However, unlike the previous study, patients categorised into the cluster with generalised extra-renal disease (cluster 2) did not have significant renal involvement in our study. Additionally, patients with cardiovascular disease and gastro-intestinal disease did not segregate into a different cluster in the present study. Patients with limited disease (cluster 1) comprised the largest group while renal AAV with low extra-renal disease was the major group in trial patients. These differences may be due to the different settings of the two studies. Moreover, we restricted our study to patients with GPA while the former study had included patients with MPA and EGPA in addition to patients with GPA. Gastrointestinal and cardiovascular involvement comprises severe disease and is more common in patients with MPA and EGPA, and hence, these patients may have resulted in the clusters observed in the trial, while they were not included in our study.

AAV has been empirically subdivided into 5 categories, i.e. localised, early systemic, generalised, severe renal and salvage therapy, by the European therapeutic trial in AAV group on the basis of medical therapy used in clinical trials [3]. However, a formal data driven classification of GPA does not exist. The categorisation of patients into prognostically relevant distinct clusters may help to improve understanding of prognosis. The evolution of patients in cluster 1 to a more generalised phenotype may indicate the need for more intensive therapy at diagnosis and questions the current approach to treat this phenotype as mild disease. A formal classification system based on multiple presenting features is likely to be better than stratification based on involvement of single specific organs such as renal, cardiovascular or gastrointestinal involvement. Hence, the results of this study may serve as an important guide to stratify GPA patients, particularly in clinical trials.

Analysis of comorbidities within clusters was descriptive only. Some of the difference in comorbidity distribution may be attributable to the age of individuals within the clusters rather than duration of GPA diagnosis. At baseline, median age increased across the clusters, cluster 1 being the youngest and cluster 3 the oldest; many comorbidities were more prevalent in the older clusters at baseline. Trends in comorbidities were similar across the three clusters, with a steady increase over the 3 years. The increase in comorbidities is correlated with increasing median age in clusters 1 and 3 over the 3-year period, and the association between comorbidities and age in GPA is well established [19, 20]. However, median age remained relatively constant in cluster 2 as the result of a substantial proportion of patients being lost to follow-up or dying (cf. Supplementary table 1). Despite this, comorbidities also increased in cluster 2 over time, suggesting that not all of the increase is due to ageing of the population.

### Strengths and limitations

The strengths of this study are the generalizability of the results due to a population-based setting, and adequate sample size. In addition, we used a robust methodology of latent class analysis for identifying disease clusters, which is highly suitable for the sample size of this study, and the high chance of missing data due to the primary care setting and use of routinely collected data. This method automatically fixes the sample size based on the true difference in the variables, and hence, prior assumptions have minimal bearing on results.

The study has some limitations. The small number of patients in cluster 3 renders the results of this subgroup less definitive. Due to the primary care setting of the patients, the results of ANCA serology were not included and the clusters were purely based on clinical phenotype. Recent changes to the classification of GPA have included PR3-ANCA [21], and previous studies have suggested clinical outcomes and genetic susceptibility are better predicted by ANCA status rather than GPA or MPA disease type [22, 23]. Nevertheless, the positivity for PR3 ANCA in GPA is 70–80%, and all patients included in our study had been diagnosed with GPA hence the omission of serology may not have had significant impact on classification, unlike the scenario for AAV as a whole [2]. ANCA itself is not diagnostic of GPA. Data on exact dose of steroids was not available. However, the duration of steroid use was estimated by calculating the number of 3-monthly intervals during which patients received a prescription of steroids.

## Conclusions

In conclusion, cluster analysis of patients with GPA presenting to primary care practices, based on information routinely collected in the electronic medical record, revealed three distinct clusters with different prognosis, susceptibility to recurrent infections and presence of comorbidities. This classification may be useful in routine practice to guide general practitioners to prognosticate patients.

## Supplementary Information

**Additional file 1: Supplementary table 1**: Evolution of symptoms in GPA clusters during 1 and 3 years’ follow-up (number of patients who newly developed symptoms up to 1 year and 1-3 years after index date).

**Additional file 2: Supplementary table 2**: Baseline demographic characteristics of patients and mortality in cluster 1 (with GPA) and a matched control population without GPA.

## Data Availability

The data that support the findings of this study are available from IQVIA but restrictions apply to the availability of these data, which were used under license for the current study, and so are not publicly available. Data are however available from the authors upon reasonable request and with permission of IQVIA.
