# Supplementary material for: Cluster analysis of patients with granulomatosis with polyangiitis (GPA) based on clinical presentation symptoms: a UK population-based cohort study
Source: Arthritis Res Ther. 2022 Aug 19;24:201. doi: 10.1186/s13075-022-02885-9 (PMC9389785; doi:10.1186/s13075-022-02885-9)
Supplement: Supplementary file 1 — Additional file 1: Supplementary table 1: Evolution of symptoms in GPA clusters during 1 and 3 years’ follow-up (number of patients who newly developed symptoms up to 1 year and 1-3 years after index date). [file 13075_2022_2885_MOESM1_ESM.docx]

**Supplementary table 1: Evolution of symptoms in GPA clusters during 1 and 3 years’ follow-up (number of patients who newly developed symptoms up to 1 year and 1-3 years after index date)**

| **New onset symptoms** | **Cluster 1** | **Cluster 2** | **Cluster 3** |
| --- | --- | --- | --- |
| Patients with 1 year of follow-up, N | 426 | 176 | 47 |
| Median (IQR) age in population at 1 year follow-up, years | 59.5 (48.8-68.2) | 64.5 (54.8-75.1) | 69.9 (58.7-80.2) |
| Patients with 3 years’ follow-up, N | 249 | 67 | 43 |
| Median (IQR) age in population at 3 years’ follow-up, years | 60.6 (50.6-68.5) | 64.0 (55.0-72.1) | 71.5 (60.8-76.3) |
| Eye, n  At 1 year  At 3 years | 81  0 | 21  0 | 6  0 |
| ENT, n  At 1 year  At 3 years | 110  13 | 11  02 | 11  01 |
| Cough, n  At 1 year  At 3 years | 124  16 | 14  6 | 5  2 |
| Breathlessness, n  At 1 year  At 3 years | 79  10 | 18  4 | 6  0 |
| Cardiovascular (chest pain/angina), n  At 1 year  At 3 years | 23  1 | 4  0 | 1  0 |
| Chest pain (not specified), n  At 1 year  At 3 years | 94  6 | 18  3 | 8  0 |
| Chronic kidney disease, n  At 1 year  At 3 years | 25  28 | 12  9 | 1  2 |
| Acute renal failure, n  At 1 year  At 3 years | 37  0 | 13  0 | 0  0 |
| Proteinuria/haematuria, n  At 1 year  At 3 years | 32  8 | 13  2 | 1  1 |
| Fatigue, n  At 1 year  At 3 years | 73  14 | 20  0 | 4  0 |
| Constitutional, n  At 1 year  At 3 years | 18  1 | 20  0 | 0  0 |
| Musculoskeletal, n  At 1 year  At 3 years | 85  3 | 24  0 | 1  0 |
| Neuropathy, n  At 1 year  At 3 years | 22  4 | 13  3 | 1  0 |
| Dermatological (rash), n  At 1 year  At 3 years | 105  4 | 22  3 | 7  1 |
| Gastrointestinal, n  At 1 year  At 3 years | 69  0 | 15  0 | 5  0 |
| Non-specific abdominal symptoms, n  At 1 year  At 3 years | 76  7 | 16  3 | 6  1 |
