# Supplementary material for: Cluster analysis of patients with granulomatosis with polyangiitis (GPA) based on clinical presentation symptoms: a UK population-based cohort study
Source: Arthritis Res Ther. 2022 Aug 19;24:201. doi: 10.1186/s13075-022-02885-9 (PMC9389785; doi:10.1186/s13075-022-02885-9)
Supplement: Supplementary file 2 — Additional file 2: Supplementary table 2: Baseline demographic characteristics of patients and mortality in cluster 1 (with GPA) and a matched control population without GPA. [file 13075_2022_2885_MOESM2_ESM.docx]

**Supplementary table 2: Baseline demographic characteristics of patients and mortality in cluster 1 (with GPA) and a matched control population without GPA**

|  | **Cluster 1 (Limited disease) (n=426)** | **Matched Control population**  **(n=4260)** |
| --- | --- | --- |
| **Number of patients** | 426 | 4260 |
| **Median (IQR) age, years** | 58.47 (47.8- 67.3) | 58.33 (47.3-67.2) |
| **Gender Females (%)** | 245 (57.5) | 1810 (42.5) |
| **Smoking history, n (%)**  **Missing** | 186 (47.3)  33 (7.7) | 1785 (41.9)  384 (9.0) |
| **Townsend quintile, n (%)**  **1 (lowest)**  **2**  **3**  **4**  **5**  **Missing** | 107 (25.1)  83 (19.5)  83 (19.5)  63 (14.8)  33 (7.7)  57 (13.4) | 945 (22.2)  853 (20.0)  784 (18.4)  574 (13.5)  448 (10.5)  656 (15.4) |
| **No. of deaths (%)** | 52 (12.2%) | 345 (8.1%) |
| **Person-years, median (IQR)** | 5.14 (2.00 - 8.69) | 5.52 (2.64 - 9.20) |
| **Unadjusted HR (95% CI), p value** | 1.65 (1.20-2.20), p<0.01 | |
| **Adjusted HR (95% CI), p value*** | 1.68 (1.16-2.42), p<0.01 | |

* Adjusted for age, sex, smoking history and Townsend quintile.
